# Final-year logbooks in otorhinolaryngology in Germany – insights from a needs analysis and user experience study of final-year students and supervisors

**DOI:** 10.3205/zma001871

**Published:** 2026-09-15

**Authors:** Kathrin Gerstacker, Jonas Engert, Luke Lükewille, Adrian von Witzleben, Lisa Budelmann

**Affiliations:** 1 University Medical Center Freiburg, Department of Otorhinolaryngology, Freiburg, Germany; 2 University Hospital Würzburg, Department of Otorhinolaryngology, Würzburg, Germany; 3 University Hospital Schleswig-Holstein, Department of Otorhinolaryngology, Kiel, Germany; 4 Ulm University Hospital, Department of Otorhinolaryngology and Head and Neck Surgery, Ulm, Germany; 5 University Medical Center Hamburg-Eppendorf, Department of Otorhinolaryngology, Hamburg, Germany

**Keywords:** digitalization, logbook, interactive, otorhinolaryngology, final-year medical education

## Abstract

**Background:**

In an effort to improve the structured training during the practical year in Germany, the German Medical Licensing Regulations have made it mandatory for final-year students to keep a logbook in 2013. This strategy aims to ensure the relevance and review of learning content and to improve the transparency and quality of teaching in the final year. The logbook recommended by the German Society of Otorhinolaryngology is to be used nationwide.

**Methods:**

Our study investigated the acceptance and use of logbooks through voluntary, anonymous online surveys of supervisors and students in Germany. The questionnaires contained eight and twelve questions, respectively. A total of 50 supervisors and 28 students participated in the survey.

**Results:**

The results showed that 26% of supervisors and 21% of students do not use a logbook. 64% of the supervisors preferred a digital version of the logbook, while 60.7% of the students also mostly agree with a digital version. It is particularly noteworthy that 79% of the students, who currently rarely use the logbook (as an analog version), stated that they would use it more often if it were available digitally.

**Conclusion:**

Our study confirms the highly heterogeneous use of logbooks during the practical year in otorhinolaryngology. Faculty-owned logbooks can be customized to specific curricula and needs more easily, which explains their popularity. Although most supervisors consider digitization useful, there are concerns about the effort involved and the potential lack of added value. Therefore, a digitized logbook should be designed not only as a control tool, but also as an interactive learning companion with clear learning objectives, in order to promote internal learning motivation.

## Zusammenfassung

**Hintergrund:**

Um die strukturierte Ausbildung im Praktischen Jahr (PJ) in Deutschland zu verbessern, wurde mit der Novellierung der Approbationsordnung für Ärzte im Jahr 2013 die verpflichtende Nutzung eines PJ-Logbuchs eingeführt. Ziel dieser Maßnahme ist es, die Vermittlung und Überprüfung relevanter Lerninhalte sicherzustellen sowie die Transparenz und Qualität der Lehre im Praktischen Jahr zu verbessern. Hierfür wird die bundesweite Verwendung des von der Deutschen Gesellschaft für Hals-Nasen-Ohren-Heilkunde entwickelten PJ-Logbuchs empfohlen.

**Methoden:**

Unsere Studie untersuchte die Akzeptanz und Nutzung von Logbüchern mittels freiwilliger, anonymer Online-Befragungen von Lehrverantwortlichen und Studierenden in Deutschland. Die Fragebögen umfassten acht beziehungsweise zwölf Fragen. Insgesamt nahmen 50 Lehrverantwortliche und 28 Studierende an der Befragung teil.

**Ergebnisse:**

Die Ergebnisse zeigten, dass 26% der Lehrverantwortlichen und 21% der Studierenden kein Logbuch verwenden. 64% der Lehrverantwortlichen und auch 60,7% der Studierenden bevorzugten eine digitale Version des Logbuchs. Besonders hervorzuheben ist, dass 79% der Studierenden, die das Logbuch derzeit selten nutzen (als analoge Version), angaben, es häufiger zu verwenden, wenn es digital verfügbar wäre.

**Schlussfolgerung:**

Unsere Studie bestätigt die hohe Heterogenität bei der Nutzung von Logbüchern im Praktischen Jahr der Hals-Nasen-Ohren-Heilkunde. Fakultätseigene Logbücher lassen sich leichter an spezifische Curricula und lokale Anforderungen anpassen, was ihre weite Verbreitung erklärt. Obwohl die Mehrheit der Lehrverantwortlichen die Digitalisierung als sinnvoll erachtet, bestehen Bedenken hinsichtlich des damit verbundenen Aufwands und eines möglichen fehlenden Mehrwerts. Ein digitales Logbuch sollte daher nicht ausschließlich als Kontrollinstrument konzipiert sein, sondern auch als interaktiver Lernbegleiter mit klar definierten Lernzielen, um die intrinsische Lernmotivation zu fördern.

## Introduction

Since 2013, the use of subject-specific logbooks in the final year has been mandatory in accordance with the German Medical Licensing Regulations (ÄAppO). The aim of this measure is to structure teaching and thus optimize learning success and exam preparation. In the same year, the German Society for Otorhinolaryngology, Head and Neck Surgery (DGHNO-KHC) developed a paper-based logbook with the recommendation for nationwide use, whereby faculty-specific additions or adaptations were explicitly desired.

In addition to the clear advantages of a standardized structure offering comparable conditions for all students across Germany, as well as uniform minimum standards and guidelines for both students and teaching staff, there are also many arguments in favor of standardized logbooks from a medical education perspective. For example, by integrating competencies and formative assessments, constructive alignment can be achieved within the clinical placement, which continues right through to the third state examination [1]. The structured feedback mechanisms established in this way can also enhance the t-value and learning outcomes for students [2], as well as their self-confidence [3]. Furthermore, a well-designed and thoughtfully structured logbook could also enhance students’ self-management skills by enabling them to work through the material at their own pace and in their own way, and to seek feedback on their progress independently.

An initial evaluation of the use of the provided logbook following its introduction in 2013 was conducted by Offergeld et al. in 2020 [4]. This study found a major acceptance of the analogue logbook among supervisors and final-year students in the field of otorhinolaryngology (ENT). However, despite being an intrinsic part of our private lives, digitalization is not yet widely adopted in medicine, neither in everyday clinical practice nor in medical training [5], [6].

Over ten years have passed since the introduction of the mandatory logbook, and the framework conditions have changed fundamentally. Rapid digital developments, the potential application of artificial intelligence (AI), the pandemic’s role in driving numerous new digital concepts and the evolving needs of a new digital generation of students all necessitate a reevaluation of the existing ENT logbook. Alongside the new digital reality, a number of developments have occurred in medical education and the demands placed on final-year students. Notably, the incorporation of entrustable professional activities and a graduate profile in the National Competence-Based Learning Objectives Catalogue for Medicine 2.0 (NKLM 2.0) ([https://www.nklm.de/], graduate profil), as part of the political efforts to reform the licensing regulations for human medicine, underscores the necessity of revising the current logbook.

This scientific work was driven by three key hypotheses and aims to enhance the existing logbook and inform the future teaching of final-year students. Firstly, we suspected that the current use of the logbook was inconsistent and not universal. Although its use was made mandatory by licensing regulations in 2013, the Teaching and Examination Working Group of the Society of Otorhinolaryngology has observed a growing preference for new faculty logbooks in recent years.

Secondly, we assumed that the main reason for the logbook’s low usage was its analogue format. While digitalization has advanced in almost all areas of life, medical education lags behind these developments in many respects. We hypothesized that users would no longer find an outdated, rigid, paper-based system suitable for their needs and expectations, having become accustomed to personalized, adaptable, interactive platforms [7]. 

This leads to the third hypothesis, which assumes that the current generation of students, who are digital natives, has a strong preference for digital applications. Having grown up with digital technologies, these students expect efficient, user-friendly, flexible solutions that optimize their education. To meet the demands of this new generation of students and improve the quality of teaching, the logbook must be converted into a digital format.

## Material and methods

### Study procedure

We designed an anonymous, voluntary, multicenter online survey for each target group. The questionnaire for supervisors consisted of eight questions (see table 1 (Tab. 1)), while the questionnaire for final-year students contained twelve questions (see table 2 (Tab. 2)). Both questionnaires both were distributed nationwide in Germany.

The online survey for supervisors consisted of a first section with three questions to record the current logbook situation at the respective clinic, followed by a second section with five questions asking about the digitization status of the logbook. The student survey comprised a first section with six questions on the general logbook situation and use at the respective clinic and a second section with six questions on the status and wishes regarding the digitization of the logbook.

The questionnaires were created using the evaSys survey portal (Electric Paper Evaluation Systems) of the Julius-Maximilians-Universität Würzburg. A total of 59 supervisors (pre-registered as teaching coordinators at the DGHNO-KHC) from all ENT university clinics in Germany were contacted via email and asked to participate in the survey. They were also asked to forward the survey to their students. The objectives and data protection conditions of the study were communicated transparently to all participants and informed consent to participate was obtained from all participants. The questionnaire was online for a total of 17 weeks. Halfway through the study period (after eight weeks), there was a change in the tertial and thus also a change in students. In addition to the initial invitation email, two reminder emails were sent out after eight and twelve weeks, respectively. 

### Statistical analysis

Statistical analysis of the data was performed using version 4.0.0 of the R statistical program (R Foundation for Statistical Computing, Vienna, Austria). The results were calculated using the median, standard deviation, minimum and maximum.

### Ethical approval

All of the described studies were conducted with the approval of the responsible ethics committee at the University of Würzburg and the responsible data protection officer, and in accordance with national legislation and the current revised version of the Declaration of Helsinki. The data was collected anonymously, so it was not possible to draw conclusions about the participants. Informed consent to participate was obtained from all participants in the study. According to the ethics committee, the study did not require an ethics vote as it fell outside the scope of section 15 of the professional code of conduct.

## Results

The questionnaires were sent out with a response time of 121 days. Of the 59 supervisors contacted, 52 completed the survey, which corresponds to a response rate of 88%. Two questionnaires from supervisors could not be considered as the respective persons did not belong to the specialty of ENT medicine (question 1.1), so 50 questionnaires from supervisors were evaluated. In addition, the online questionnaire was answered validly by 28 students. 

### Survey 1 – supervisors 

#### Current logbook

Of the total of 50 supervisors, 37 (74%) stated that they use a logbook at their ENT clinic. In total, 13 participants (26%) stated that they do not use a logbook (question 1.2). When asked about the type of logbook used in the clinic, nine supervisors (18%) stated that they use the logbook of the German Society of Otorhinolaryngology (DGHNO-KHC), while 41 participants (82%) use an internal faculty logbook (question 1.3).

#### Need for digitization

Of the 50 participants, 32 (64%) said they preferred a digital logbook to an analogue one (question 1.4). Meanwhile, 26 respondents (52%) agreed that there was sufficient infrastructure for a digital logbook at their clinic (question 1.5). When asked whether they considered the introduction of a digital logbook to be meaningful, 37 of supervisors (74%) agreed, while 13 (26%) disagreed (question 1.6). When asked in which format they would find a digital logbook appealing (question 1.7), 33 participants (66%) opted for an app, six (12%) for an intranet-based version, eight (16%) for a digital file (Word or PDF), and three (6%) selected the “other” option.

A comparison of the desire for a digital logbook (question 1.4) and the currently used logbook (question 1.3) showed that of the nine supervisors who use the logbook provided by the society of their clinic, six (66.7%) would prefer a digital version, while three (33.3%) would like to retain an analogue format. Of the 41 supervisors who use a faculty-specific logbook, 28 (68.3%) stated that they would like a digital logbook. A total of 32 supervisors (64%) stated that they preferred a digital logbook to an analogue form (question 1.4). The results are presented in figure 1 (Fig. 1).

The free-text question 1.8 “What would you like to see in a digital logbook?” was answered by 22 (44%) participating supervisors (see table 3 (Tab. 3)). The main items mentioned were accessibility and availability, individualization, simplicity, and standardization. However, skepticism was also mentioned.

### Survey 2 – students

#### Current logbook

Of the 28 ENT students who participated in the study, 22 (79%) stated that they use a logbook, while six students (21%) stated that they do not. Four students (14%) use the logbook of the German Society of Otorhinolaryngology, while 24 students (86%) use a faculty logbook. In response to question 2.4 of the student survey, which asked about the frequency of use of a digital logbook, 17 participants (61%) answered positively and eleven students (39%) answered negatively (see figure 2 (Fig. 2)).

When asked about the reasons for using the logbook, which allowed multiple answers, the participants stated “out of obligation” 13 times, “to get an overview of expected performance” seven times, “as a clinical activity guide” three times, and “to coordinate learning progress” five times. No participant stated that they were already using a digital version. Almost two thirds of the students surveyed (68%) said that they rarely or never use the logbook. Only 7% said they used it all the time (see figure 3 (Fig. 3)).

#### Need for digitization

Of the 28 ENT students who participated, 17 (61%) stated that they preferred a digital format to an analogue one. Regarding the specific format, 20 (71%) students voted for an app as their favored format, two (7%) for an intranet-based application, four (14%) for another version and two (7%) for a digital file (Word or PDF) (question 2.11). The results are presented in figure 2 (Fig. 2).

The question as to what content should definitely be included in a digital logbook for final-year students in ENT was answered by eight participants (29%), highlighting clear learning objectives, accessibility, guiding structures and mentoring in particular (question 2.12, see table 4 (Tab. 4)). 

A comparison of students using the basic logbook provided by the specialist association with those who use a faculty's own version shows that 80% of users of the basic logbook are in favor of a digital application. By contrast, only 48% of students with a faculty logbook said they would prefer a digital version.

Of the 19 students who stated that they currently never or rarely use the logbook (question 2.9, see figure 3 (Fig. 3)), 15 students (79%) desired a digital version. Of the nine students who stated that they frequently or always use the logbook, only two participants (22%) agreed with the wish for a digital version.

## Discussion

Since 2013, keeping a logbook for students in their final year has been mandatory according to the ÄAppO intending to ensure the relevance and review of the learning content, and thus achieve an improvement and transparency of teaching in the final year. On the recommendation of the Medical Faculty Association (MFT), the DGHNO-KHC logbook was published in 2013 as a nationwide recommendation to ensure uniform application and quality assurance. Offergeld et al. (2020) found that 36% of German ENT clinics reported issuing a faculty-owned logbook, with 73% of students using one [4]. 

Four years later, our study shows that a faculty-own logbook is used in over 82% of cases (82% of supervisors and 86% of students). There is a clear trend towards faculty-intern variants. This development could be related to the fact that faculty-specific formats allow for better differentiation, profiling and individual learning objectives or have developed due to local formalities. Barteit et al. identified this as a decisive element for implementation [8]. Another possible reason for the need for further development of the logbooks could be the recent rapid developments in medical didactics, such as the establishment of EPAs. This is underlined by the initial results of an interdisciplinary working group at the MFT [9], [[10]. An EPA-based logbook for surgery, internal medicine and family medicine was developed and piloted in 2019. 

However, too much individualization of logbooks can lead to structural and content-related differences in the training of students, and thus also to inhomogeneous basic requirements at the start of their careers. The development of an overarching graduate profile as part of the NKLM process clarifies the goal of a uniform degree level ([https://www.nklm.de/], graduate profil). 

Studies over the past few decades have shown that the use of logbooks by both students and teachers is often severely restricted across disciplines and countries [11]. Our study confirms this, showing that despite the logbook being mandatory, a total of 26% of supervisors and 21% of students do not use one. These results support our first hypothesis that the use of logbooks is currently not universal and inhomogeneous.

A logbook is not used if it is perceived by students as boring and repetitive [12] or bureaucratic [13] and the added value for students is not apparent. If completing the logbook seems like a burden and there are no consequences for not using it [14], the usage rate will naturally fall sharply. Although constant tracking of activities and advancement is proven to be effective [13], the implementation of a digital application alone does not sufficiently lead to its usage [14], [15]. The aim of a logbook for the final year must therefore be to increase the individual intrinsic motivation to use it. 

In our study, of the 19 students who rarely or never use the logbook (question 2.9, see figure 3 (Fig. 3)), 79% stated that they would use it more often if it were digital. This supports our second hypothesis that digitalization would encourage usage. Numerous studies and reviews from other countries support this hypothesis, stating that an electronic logbook format provides better accessibility and flexibility [16], [17], allows for easier documentation and progress analysis [18], [19], and can improve interaction with a mentor [20], although acceptance of such a format should be tested beforehand. 

Today's students are digital natives. Although studies suggest that students are sometimes critical of the use of digital teaching formats [21], our study shows that this attitude does not apply to applications to the same extent. The targeted use of digital media in phases of high workload appears to be attractive and is supported by the majority of students. However, the results from question 4 on whether a digital version of the logbook would be used more frequently show that no generally valid statement can be made here either. As many as 36% of students stated that they would not use a digital version more often. This suggests that digitization alone may not be sufficient to increase usage and cannot fully confirm our third hypothesis that the new “digital natives” only want to work digitally. Emphasis should rather be put on the choice of individual aspects of a logbook that would actually benefit from being digitized. After all, just as learning styles vary greatly, so do usage patterns. However, since logbooks for residency are digital in almost all federal states in Germany, introducing a digital logbook in the final year of studies is an effective way to encourage consistent usage habits. Furthermore, analyzing these usage patterns could be valuable for the long-term development of residency logbooks.

The free text comments from our study (see table 3 (Tab. 3)) suggest that students would like a logbook to be a learning companion with added value that contains clear learning objectives and expectations, offers concrete knowledge transfer and includes organizational information as well as interactive elements. In addition to more structure, students would also like to have the option of further content-related training and an interactive platform for signing off completed tasks. Availability, low-threshold access and an overview are highlighted as the main advantages of a digital application. In particular, the reviews by Schüttpelz-Brauns et al [11] on the fundamental advantages of logbooks and Paydar et al. [20] on the advantages of digital logbooks underline that the already existing value of a logbook can be enhanced by digitization. 

Interestingly, students who already use analogue logbooks regularly seem to need less convincing to digitize them, as only 48% of respondents stated that they prefer a digital format. These students also appear to have a high level of motivation to use the analog version. On the one hand, this could be due to the individual personality structure of the students. On the other hand, there might be special formats and characteristics of specific logbooks that are more likely used than others. As we did not record the faculty of the students in our survey to preserve anonymity, only an analysis of all logbooks for ENT, available throughout Germany, can provide further information on these aspects. This has to be addressed in further studies. 

In addition to the students, the use of the logbook by teachers is also an essential part of the learning process and must be considered when developing new formats. Our study found that 64% of supervisors are in favor of a digital logbook, while 36% are against it. In the free text comments, some supervisors expressed uncertainty about a digital logbook. While overview, accessibility and structure were mentioned as advantages, it was criticized that the development and usage effort was high and the added value unclear. Some comments indicated that an app could make work more difficult, as an analogue signature is perceived as easier. This information correlates with the findings from other studies on the piloting and establishment of e-logbooks. Gondal et al. were able to show, for example, that the use of a digital logbook was predominantly rated positively among supervisors, but that here too, uncertainties in dealing with the platform and skepticism regarding the time required were expressed [22]. While the uncertainties with new applications can be quickly eliminated through training, the basic motivation of doctors to accompany students in their learning process seems to be limited, regardless of the form of the logbook. A multi-year study by Kadmon et al. [23] also showed that medical supervision of students was very low due to long surgery times in particular. When developing a new logbook format, the overarching problem of intrinsic motivation for use must therefore be considered not only for students but also for teachers. By enabling a better analysis of individual and collective learning progress at a clinic, an incentive system could be created that can be used both for qualification purposes and for scientific purposes. 

Considering the limitations of this study, there is a particular shortcoming in the response rate among students.

While the response rate of 88% in the survey of supervisors can be regarded as representative, the participation of students is quite low. 

To better understand the number of participants (n=28 medical students) who took part in the survey, an approximate extrapolation was made of the nationwide number of final-year students in the ENT elective term: Each year, around 8,000 medical students in Germany begin their final year, spread over two starting dates (May and November). Each final year comprises two compulsory terms (internal medicine and surgery) and one elective term, which can be chosen from around 35 different clinical specialties, including otorhinolaryngology (ENT). Assuming that 2-5% of students choose the elective ENT term, which corresponds to the observed demand for smaller clinical subjects, this results in an estimated number of around 160-400 PJ students per year in ENT nationwide. Since the selected survey period covers only two-thirds of a year, the number of students who can be reached is reduced to 107-267. Given this, the number of 28 participating students corresponds to an estimated response rate of approximately 10-26% of the eligible target group nationwide. Unfortunately, no data was available for final-year students, broken down by specialty. Given the voluntary nature of participation, the heterogeneity of the locations and the limited accessibility within the final-year context only via their supervisors, this rate can be considered solid. 

Another important point to consider when evaluating student feedback is the possibility of selection bias, since the link was distributed exclusively by their supervisors. Nevertheless, future studies should aim at a higher response rate by directly addressing students, for example via social media or students associations, in order to obtain an optimised representation of the student body.

## Conclusion

Digitalization in clinical practice is developing rapidly. A digital application for training students appears to be in keeping with the times and must promote intrinsic motivation to use it while conserving supervisors’ time. It is crucial that we move away from a sense of duty as the reason for use. There is hardly any discrepancy between the students and the supervisors with regard to the low usage of the logbook in general. Faculty logbooks are used frequently and provide a good source for analysis and further development. In our view, the next sensible and necessary step towards a logbook promoting a high intrinsic using-motivation and user-friendliness in everyday clinical practice is therefore a quantitative and qualitative analysis of the faculty's own logbooks. In addition, it would be interesting to determine how it would be possible to offer a standardized logbook instead of the faculty's own logbooks without losing the advantages of the faculty’s own logbooks. Therefore, a national taskforce was established to promote this efforts. 

## Notes

### Ethics approval and consent to participate

All studies described were conducted with the approval of the responsible ethics committee of the University of Würzburg, the responsible data protection officer and in accordance with national law and the Declaration of Helsinki of 1975 (in the current revised version). The data was collected anonymously; it was not possible to draw conclusions about the participants. We obtained informed content of participation from all participants. According to the responsible ethics committee, there was no advisory competence in accordance with Section 15 of the Professional Code of Conduct and therefore no need for an ethics vote.

### Consent for publication

All participants agreed to anonymous publication of data given in the survey.

### Availability of data and materials

All data generated or analysed during this study are included in this published article.

### Funding

We would like to thank the Open Access Publication Fund of the UKE – University Medical Center Hamburg-Eppendorf – for its financial support.

### Author contributions

All authors have made significant contributions to the research and manuscript. Their specific contributions are detailed as follows:

- Conceptualization: all authors
- Methodology: all authors
- Formal Analysis: LB, KG
- Investigation: all authors
- Resources: LB, KG, JE
- Data Curation: LB, KG
- Writing – Original Draft Preparation: LB, KG
- Writing – Review & Editing: all authors
- Visualization: LB
- Supervision: LB
- Project Administration: KG, JE

All authors have read and approved the final version of the manuscript.

### Authors’ ORCIDs

- Kathrin Gerstacker: [0000-0003-0582-0684]
- Luke Lükewille: [0009-0000-8437-5886]
- Adrian von Witzleben: [0000-0001-6766-4758]
- Lisa Budelmann: [0009-0001-5327-035X]

## Competing interests

The authors declare that they have no competing interests. 

## Figures and Tables

**Table 1:**
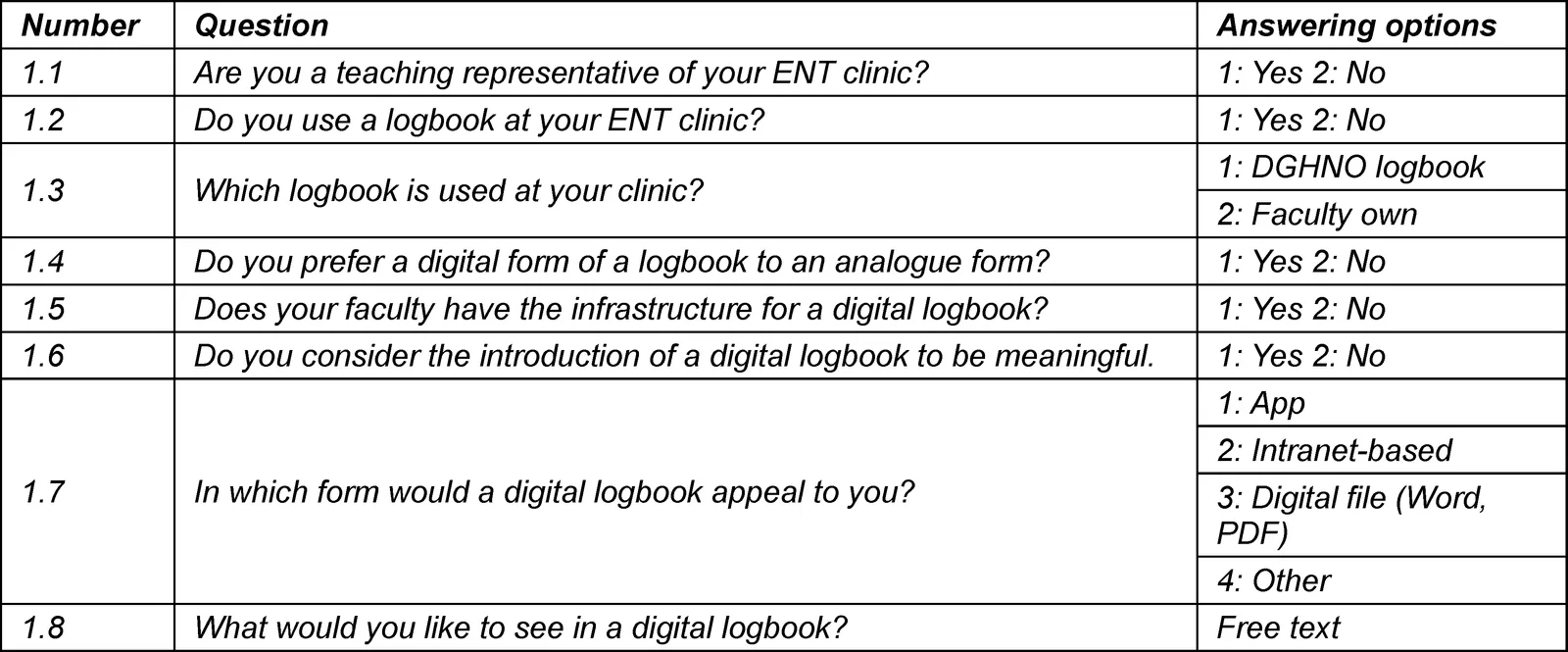
Displaying the 8 questions (1.1.-1.8) and answering options asked to supervisors within the anonymous survey

**Table 2:**
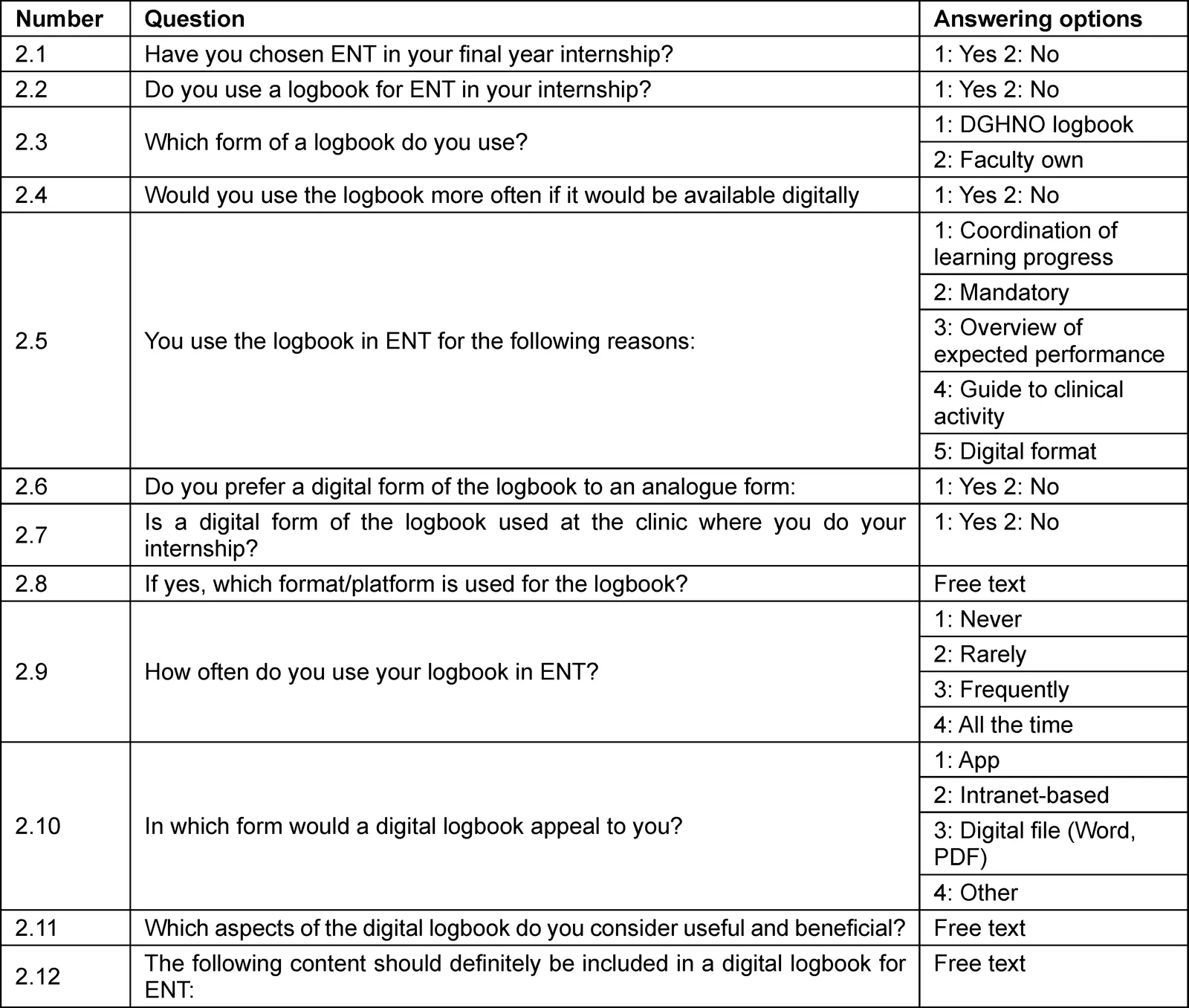
Displaying the 12 questions (2.1-2.12) and answering options asked to students within the anonymous survey

**Table 3:**
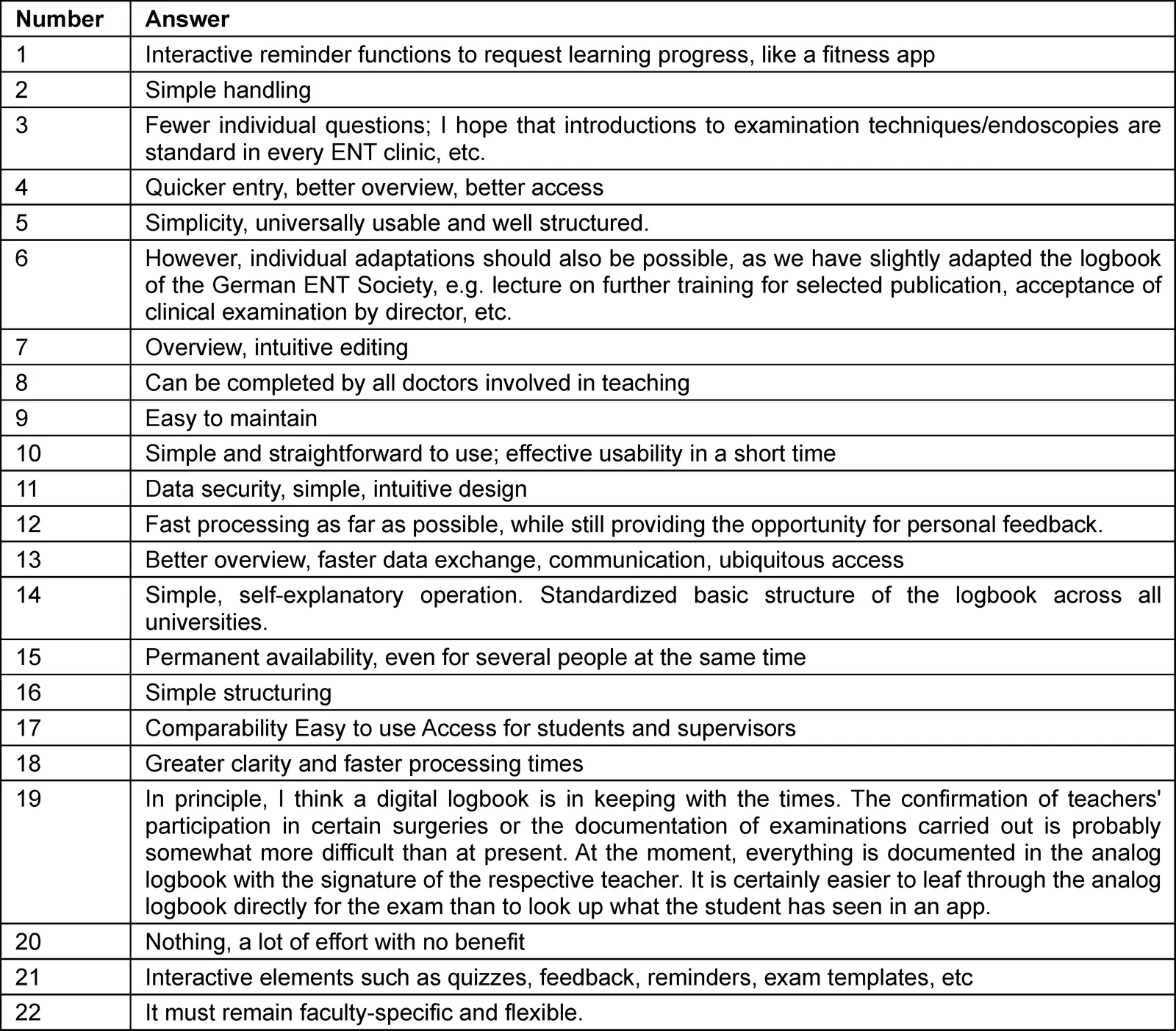
All freetext answers of supervisors regarding their wishes for a digital logbook

**Table 4:**
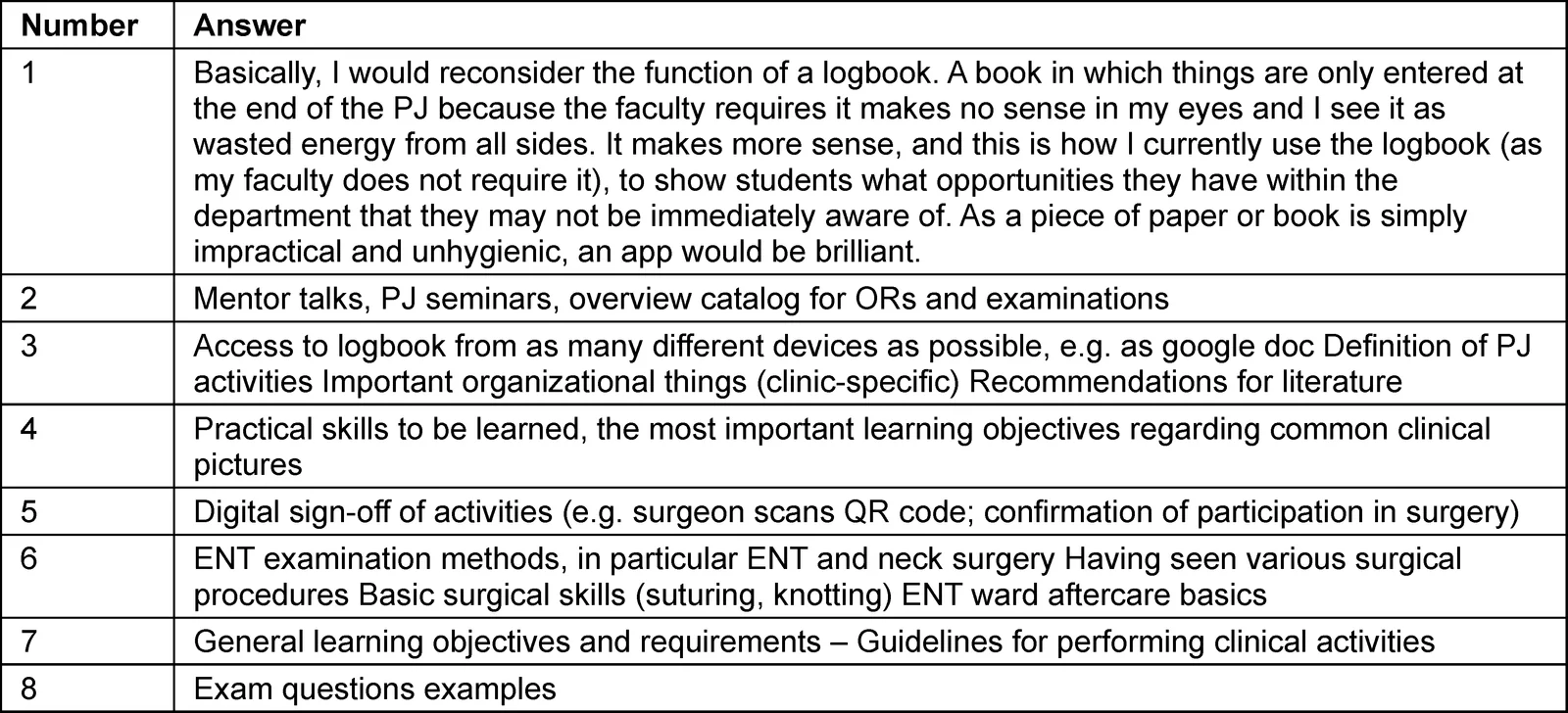
All freetext answers of students regarding their wishes for a digital logbook

**Figure 1:**
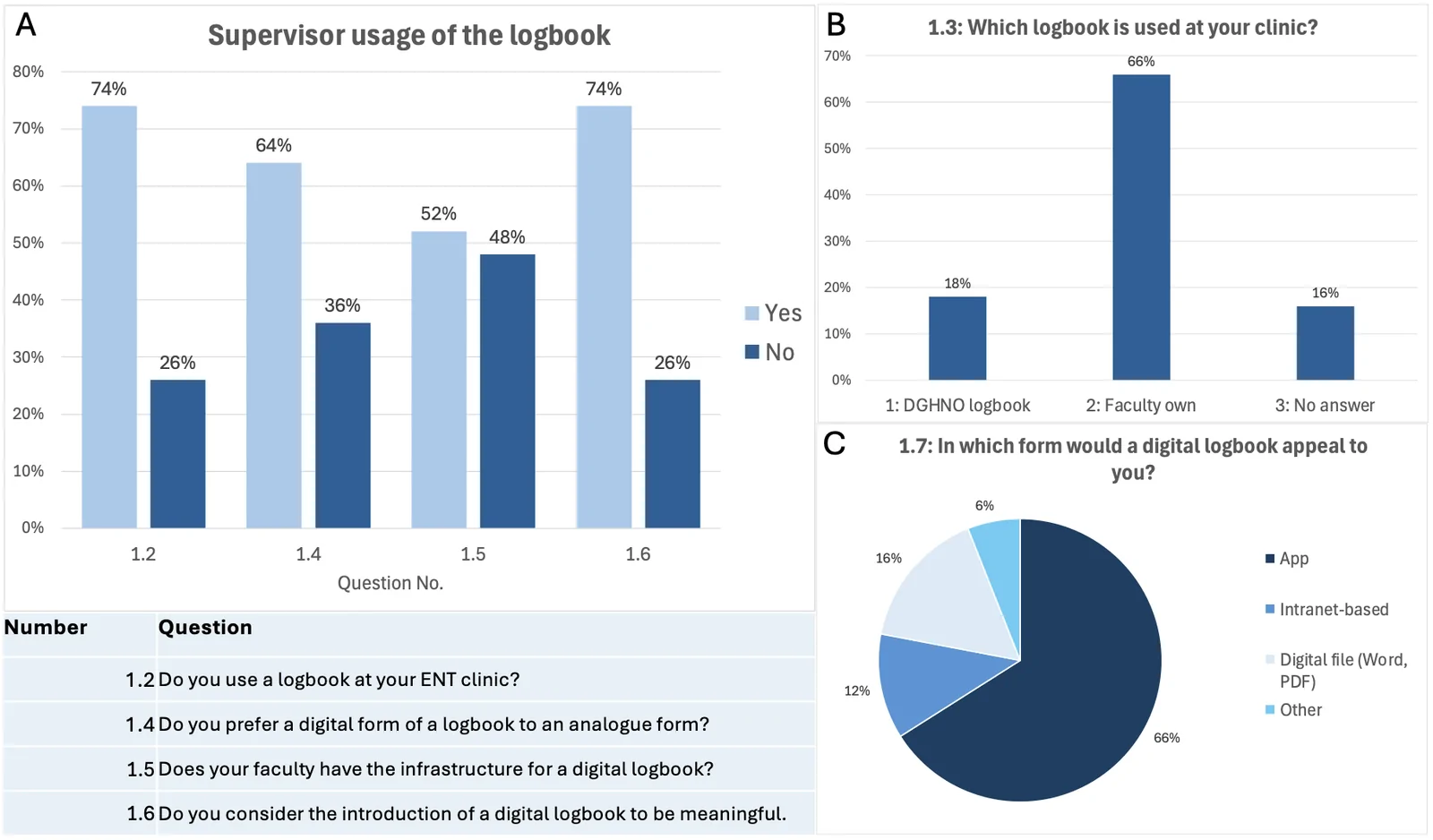
Analysis of the answers of supervisors to questions 1.2-1.7

**Figure 2:**
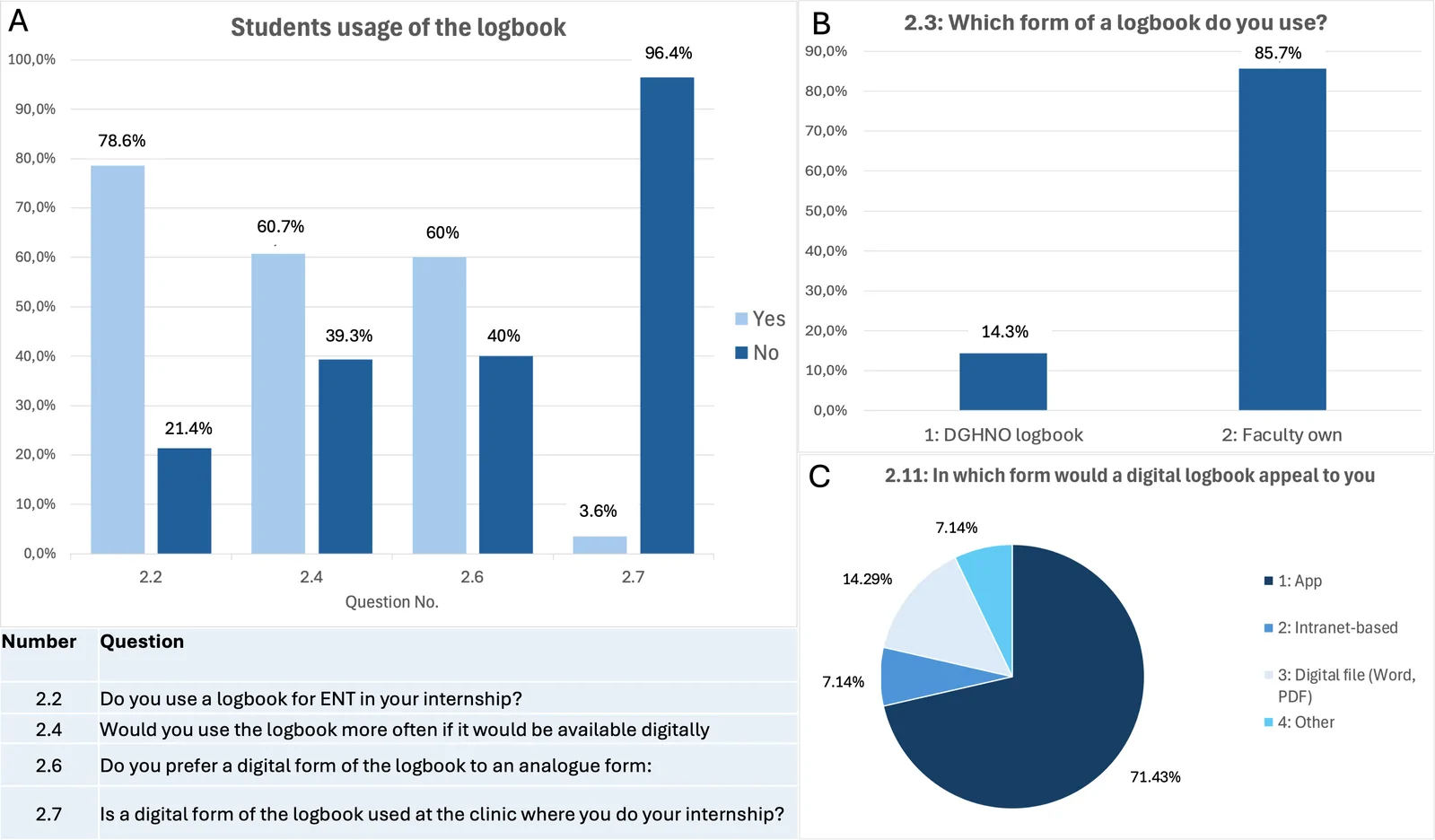
Analysis of the answers of students to questions 2.2-2.4, 2.6, 2.7 and 2.11

**Figure 3:**
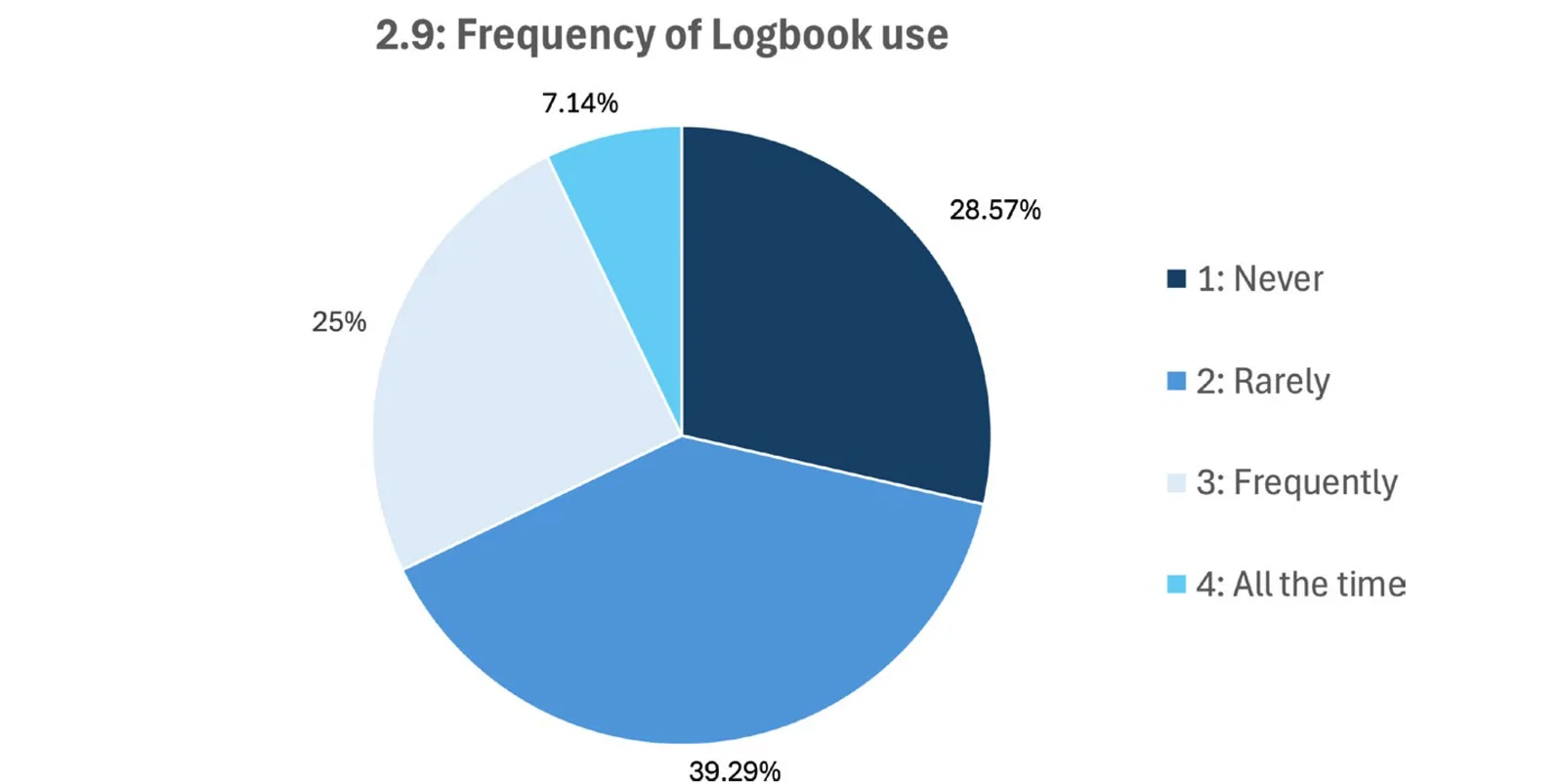
Analysis of the answers to question 2.9. regarding the frequency of logbook use of students from never to all the time during their internship
